# Immunohistological Examination of HEATR1 and SLC27A2 Expression in ccRCC Samples to Evaluate Their Potential as Prognostic Markers—A Preliminary Study

**DOI:** 10.3390/cancers17132234

**Published:** 2025-07-03

**Authors:** Michał Kasperczak, Iga Kołodziejczak-Guglas, Karolina Pawłowska-Kasperczak, Maciej Wiznerowicz, Andrzej Antczak

**Affiliations:** 1 Department of Urology, J. Struś Hospital in Poznań, Szwajcarska 3, 61285 Poznań, Poland; kara040693@icloud.com (K.P.-K.); maciej.wiznerowicz@iimo.pl (M.W.); aa26@poczta.onet.pl (A.A.); 2 International Institute for Molecular Oncology, 60203 Poznań, Poland; iga.kolodziejczak@iimo.pl; 3 University Hospital of Lord’s Transfiguration, 61848 Poznań, Poland

**Keywords:** clear cell renal cell carcinoma, HEATR1, SLC27A2, prognostic marker

## Abstract

**Background/Objectives**: Clear cell renal cell carcinoma (ccRCC) is a common kidney cancer with limited therapeutic options. This study investigated the expression of HEAT repeat-containing protein 1 (HEATR1) and solute carrier family 27 member 2 (SLC27A2) in ccRCC and their potential as prognostic markers and therapeutic targets. **Methods**: Analysis of a public proteomic dataset (CPTAC) and immunohistochemistry (IHC) validation in an independent cohort of 52 ccRCC patients was performed. HEATR1 and SLC27A2 expression were correlated with survival outcomes. Reactome pathway analysis was conducted to explore the functional roles of HEATR1 and SLC27A2. **Results**: The analysis showed that HEATR1 is upregulated and associated with poor prognosis, while SLC27A2 is downregulated and similarly linked to shorter progression-free survival. High HEATR1 expression and low SLC27A2 expression correlated with shorter progression-free survival (PFS) and overall survival (OS) in patients with high-grade ccRCC. Reactome analysis indicated HEATR1’s involvement in RNA metabolism and SLC27A2’s role in lipid metabolism, particularly peroxisomal lipid metabolism and fatty acyl-CoA biosynthesis. HEATR1 exhibited a dual localization in both the cytoplasm and nucleus, while SLC27A2 was primarily observed at the cell membrane and the nucleus. This different subcellular distribution suggests multifaceted roles for both proteins in ccRCC pathogenesis. **Conclusions**: HEATR1 and SLC27A2 are potential prognostic markers in ccRCC. Further research is needed to validate these findings in larger, more diverse cohorts and elucidate their roles in ccRCC progression.

## Simple Summary

This study investigated the expression of HEAT repeat-containing protein 1 (HEATR1) and solute carrier family 27 member 2 (SLC27A2) in clear cell renal cell carcinoma (ccRCC). The analysis showed that HEATR1 is upregulated and associated with poor prognosis, while SLC27A2 is downregulated and similarly linked to shorter progression-free survival. High HEATR1 and low SLC27A2 expression correlated with cancer progression, relapse, and overall survival in patients with high-grade ccRCC. Functional analysis suggests that HEATR1 is involved in RNA metabolism and SLC27A2 in lipid metabolism. These findings implicate HEATR1 and SLC27A2 as potential prognostic biomarkers in ccRCC.

## 1. Introduction

Clear cell renal cell carcinoma (ccRCC) accounts for approximately 3% of all cancer diagnoses, with the highest prevalence in Western countries [1,2]. In the developed countries, most cases are detected by chance during imaging tests like ultrasound, CT scans, or MRIs [3]. Only about 10% of patients experience the classic triad of symptoms—hematuria, flank pain, and palpable mass.

ccRCCs account for 75% of all kidney cancers. They develop from renal tubular epithelial cells in the proximal nephron and often spread through the bloodstream to organs like the liver, lungs, and bones [4]. Interestingly, nearly half of ccRCC cases (up to 45%) are associated with inactivating somatic mutations or deletions in the von Hippel–Lindau (VHL) tumor-suppressor gene [5]. A smaller percentage (5%) is due to germline mutations in the VHL gene (VHL disease), and people with inherited tuberous sclerosis genes are also at higher risk of developing bilateral kidney cancer before the age of 46. Factors like diet, alcohol, obesity, poorly managed hypertension, smoking, and occupational exposures can increase the risk of RCC [6]. Preventive strategies are crucial to improving survival rates and reducing health disparities. These include enhanced imaging protocols for early RCC detection and programs addressing lifestyle factors and access to healthcare, particularly among marginalized populations [7]. The challenges associated with treating advanced/metastatic ccRCC have fueled extensive research into the molecular mechanisms driving tumor progression and identifying novel therapeutic targets [8]. Among these, tumor-infiltrating immune cells (TIICs), a subset of non-cancerous cells within solid tumors, have emerged as crucial players in both tumor promotion and suppression [9]. Their significant role is underscored by their close association with immunotherapy response and clinical outcomes [10].

HEAT repeat-containing protein 1 (HEATR1) is a protein involved in ribosome biogenesis, essential for cellular protein synthesis. HEATR1 is a functional and structural homolog of yeast U3 small nucleolar RNA-associated protein 10 (UTP10) [11]. This protein belongs to the PR65/A subunit of protein phosphatase 2A and the elongation factor-3 family. While its exact role in cancer remains unclear, HEATR1 exhibits high expression in glioblastoma tissues and cytotoxic T lymphocytes of glioma patients [12]. Research has highlighted the importance of lipid metabolism in cancer development and progression [13]. Cancer cells meet their specific lipid metabolism needs through heightened de novo fatty acid (FA) biosynthesis or by absorbing exogenous FAs. Active transport via fatty acid transporters (FATPs) and fatty acid translocase (FAT/CD36) is a key pathway for cellular uptake of exogenous FAs [14,15]. FATP2, encoded by SLC27A2, is a multifunctional protein that acts as a gatekeeper in FA transport. Recent research has revealed that SLC27A2 influences intracellular lipid homeostasis and plays a vital role in type 2 diabetes, renal fibrogenesis, and tumor progression [16,17,18,19,20].

In this study, we investigated the expression levels of SLC27A2 and HEATR1 in ccRCC. Our aim is to evaluate their potential as molecular markers for predicting survival rates and to elucidate their roles in ccRCC pathogenesis. The candidate proteins were selected based on the CPTAC database results and validated using IHC performed on samples obtained from ccRCC patients.

## 2. Materials and Methods

### 2.1. Patients and Cohorts

This study utilized two cohorts. The discovery cohort, obtained from the Clinical Proteomic Tumor Analysis Consortium (CPTAC), consisted of 110 treatment-naïve ccRCC cases and 84 paired normal adjacent tissue (NAT) samples, as previously described by Clark et al. [19]. Publicly available liquid chromatography-tandem mass spectrometry (LC-MS/MS) protein expression data from this cohort, combined with an extensive literature review, guided the selection of HEATR1 and SLC27A2 for further investigation. These proteins were chosen based on their significant biological relevance and differential expression in tumor versus normal tissue (HEATR1 upregulated, SLC27A2 downregulated).

We used publicly available proteomic data from the CPTAC dataset for a thorough examination of protein markers linked to the development and spread of ccRCC. Our focus was on proteins with significant biological relevance, specifically those showing altered expression in tumors compared to normal adjacent tissues. We set a threshold for selection of fold change >1.5 or <0.67 and adjusted *p*-value < 0.05. We found that HEATR1 was significantly upregulated (fold change: 1.5), and SLC27A2 was markedly downregulated (fold change: 0.24). We then correlated the expression of these validated markers with clinical data using IHC analysis. This integrated approach combines proteomic data acquisition, marker validation, and clinical correlation, providing a deeper understanding of ccRCC pathology and progression, which could lead to future clinical applications.

A validation cohort comprised 52 ccRCC samples from patients aged 31 to 84 years. This cohort included 23 patients who were also part of the CPTAC discovery cohort. Detailed demographic and clinicopathological data (age, sex, race, tumor grade, and stage) were collected for all patients (Supplementary Table S1). During a five-year follow-up period, 26 patients developed metastases, while the remaining 26 did not during the five-year follow-up period. This study focused exclusively on adult patients with histologically confirmed ccRCC. Patients with a history of systemic treatment or additional cancers within the preceding 12 months were excluded in accordance with CPTAC ethical guidelines.

### 2.2. Immunohistochemistry and Pathology Evaluation

IHC was performed on retrospectively collected formalin-fixed, paraffin-embedded (FFPE) tissue samples. These samples were fixed in 10% neutral buffered formalin for 24 h before being embedded. Four-micron-thick sections were cut from the FFPE blocks and placed on positively charged slides.

The staining process used a Dako Autostainer Link 48 automated staining system with preprogrammed protocols and the EnVision visualization kit (Cat No. K800221-2, Dako, Agilent Technologies Inc., Santa Clara, CA, USA). We performed antigen retrieval using Dako Target Retrieval Solution (High pH, Cat No. S2367) in a PT Link Pre-Treatment Module (Dako, Agilent Technologies Inc., Santa Clara, CA, USA) at 97 °C for 20 min. Sections were incubated with primary antibodies against HEATR1 and SLC27A2 (Table 1) for 30 min at room temperature. For HEATR1, positive control tissues included human testis. For SLC27A2, liver tissues were used as positive controls. Negative controls included both omission of the primary antibody to assess non-specific background and staining of tissues known to lack expression of the target protein, i.e., tonsils.

Three independent pathologists evaluated the stained slides. The H-score method [20] was employed for the semi-quantitative assessment of the intensity and extent of staining. This method assigns a score from 0 to 300 by combining staining intensity (weak = 1, moderate = 2, strong = 3) and the percentage of cells stained at each intensity level. The H-score is calculated as follows: H-score = (% of cells stained at intensity 1 × 1) + (% of cells stained at intensity 2 × 2) + (% of cells stained at intensity 3 × 3). This calculation provides a detailed measure of the overall IHC staining within the tissue sample.

To categorize patients for further analysis, the average H-score for each protein was calculated across the 52 ccRCC samples. The average H-score was 105 for HEATR1 and 100 for SLC27A2, and these values were used as cutoff thresholds to classify patients into ‘High IHC score’ and ‘Low IHC score’ groups. For HEATR1, 25 patients were classified as high expression and 26 as low; one case was excluded due to technical issues during staining. For SLC27A2, 31 patients were in the high expression group and 21 in the low expression group (Table 2).

### 2.3. Digital Image Acquisition and Archiving

All IHC slides were digitally scanned at 20X magnification using a ScanScope AT Turbo whole slide scanner (Aperio/Leica Microsystems, Wetzlar, Germany). Images were saved in svs format and reviewed using ImageScope software (version 12.3.3) to ensure high quality. Digital images were stored on a password-protected Synology Rack Station server (RS18017xs+).

### 2.4. Statistical Analysis

The non-parametric Wilcoxon rank-sum test was used to compare protein abundance between normal and tumor tissues in the ccRCC discovery cohort. The same test was used to compare IHC scores between the High and Low IHC score groups in the validation cohort. Differences in protein abundance between these groups were assessed using the Mann–Whitney U test. Kaplan–Meier survival analysis with log-rank (Mantel–Cox) testing was performed to compare overall survival (OS) and progression-free survival (PFS) between the High and Low IHC score groups for both HEATR1 and SLC27A2. Hazard ratios (HR) and 95% confidence intervals (CI) were calculated. All statistical analyses were conducted using GraphPad Prism 10 software, with a significance level of *p* < 0.05.

### 2.5. Reactome Pathway Enrichment Analysis

To investigate the functional roles of HEATR1 and SLC27A2 in ccRCC and identify associated cellular processes, we performed a Reactome pathway enrichment analysis (resource available at: https://reactome.org/, accessed on 20 January 2025). Using protein expression data from the CPTAC ccRCC dataset, we used the Reactome Pathway Analysis tool to identify pathways significantly enriched for each protein. Pathways with a *p*-value < 0.05 were considered statistically significant and biologically relevant.

## 3. Results

### 3.1. Protein Expression Values in CPTAC ccRCC

**Figure 1:**
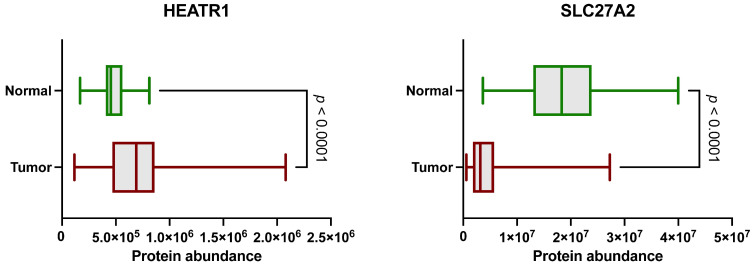
Abundance value distribution of both HEATR1 and SLC27A2 proteins in samples from the CPTAC database between normal adjacent tissue (NAT) and tumor samples. The green boxes denote NAT, and the red boxes tumor samples (*p* < 0.0001).

Analysis of publicly available LC-MS/MS data from the CPTAC cohort revealed differential expression of HEATR1 and SLC27A2 between ccRCC and normal tissue samples. Based on these initial findings, we selected HEATR1 and SLC27A2 for further analysis by examining the CPTAC database [19]. Our analysis confirmed that HEATR1 was significantly upregulated in ccRCC tissues compared to NATs (Figure 1), while SLC27A2 showed higher expression in NATs. These differences were statistically significant (*p* < 0.0001, Mann–Whitney U test).

### 3.2. Immunohistochemical Analysis of HEATR1 and SLC27A2 Reveals Potential Prognostic Markers

**Figure 2:**
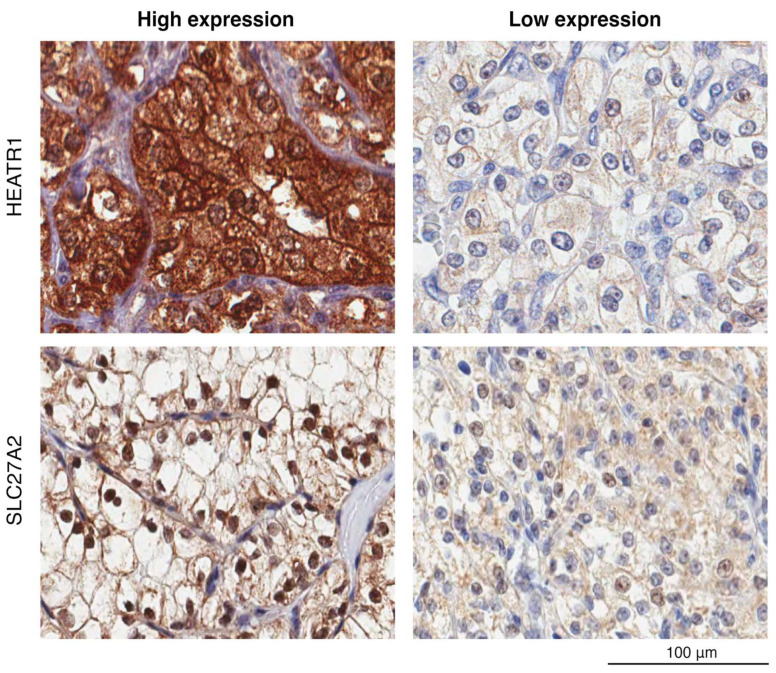
Immunohistochemical staining patterns for HEATR1 and SLC27A2 in representative ccRCC tumor sections from the validation cohort. The images demonstrate both high and low expression levels of these proteins. In the stained sections, a brown color indicates a positive immunoreaction, while a blue color signifies a negative immunoreaction.

Immunohistochemical analysis revealed distinct cellular localization patterns for HEATR1 and SLC27A2 in ccRCC cells (Figure 2). HEATR1 was expressed in the cytoplasm and nucleus, while SLC27A2 was expressed in the cell membrane of the tumor cells. Interestingly, nuclear staining of SLC27A2 was observed in a subset of tumor samples. While this finding raises the possibility of a previously unrecognized nuclear role for SLC27A2 in cancer cells, it should be interpreted with caution. The nuclear localization could also reflect a technical artifact or non-specific staining due to the use of a polyclonal antibody, particularly in the absence of staining data from non-malignant tissues or orthogonal validation. Further investigation using alternative detection methods will be necessary to clarify this observation. Following IHC staining, H-scores were generated for each protein as described in the Section 2. These scores were then correlated with patient survival data to explore the potential prognostic value of HEATR1 and SLC27A2 expression levels, particularly in relation to metastasis. Suitable known positive and negative controls were run for each antibody separately.

### 3.3. HEATR1 and SLC27A2 Expression as Measured by IHC

Following the IHC assay, analysis of H-scores for HEATR1 and SLC27A2 in the ccRCC validation cohort revealed distinct expression patterns. The observed differences in H-scores between the High and Low expression groups were statistically significant for both proteins, indicating a clear dichotomy in their expression levels. Notably, neither HEATR1 nor SLC27A2 exhibited significant outliers within their respective High and Low expression groups, suggesting consistent expression patterns within each group.

### 3.4. HEATR1 and SLC27A2 Expression Correlated with Clinical Outcomes

**Figure 3:**
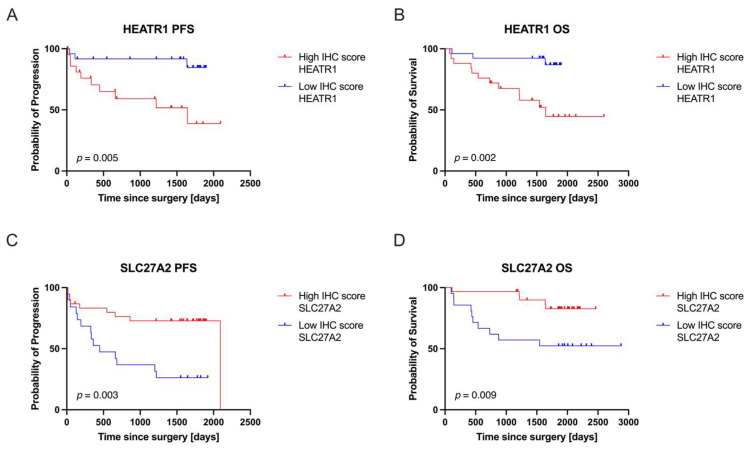
The IHC scores for HEATR1 (**A**,**B**) and SLC27A2 (**C**,**D**) are correlated with patient clinical outcomes, namely progression-free survival (PFS) and overall survival (OS). This analysis involved dividing patients into groups based on their highest and lowest IHC scores to clearly compare their outcomes, which were meticulously monitored over a five-year follow-up period.

To evaluate the clinical relevance of HEATR1 and SLC27A2 expression levels, we performed survival analyses. Patients were stratified into High and Low expression groups based on their IHC scores (Figure 3), and OS and PFS were compared over a five-year follow-up period (Table 3).

Our analysis revealed that high HEATR1 expression was significantly associated with poorer PFS (*p* = 0.005, HR = 5.141) and OS (*p* = 0.002, HR = 5.636), indicating an increased risk of disease progression and death. Conversely, low SLC27A2 expression correlated with poorer PFS (*p* = 0.003, HR = 3.299) and OS (*p* = 0.009, HR = 3.798), suggesting that decreased SLC27A2 levels are also associated with an unfavorable prognosis. Importantly, for SLC27A2, an HR > 1.0 indicates a higher risk of disease progression and death, which is observed in the low expression group. This highlights the inverse relationship between SLC27A2 expression and patient outcomes in our study.

### 3.5. Reactome Pathway Enrichment Analysis of HEATR1 and SLC27A2

To gain a deeper understanding of the functional roles of HEATR1 and SLC27A2 in ccRCC, we performed a Reactome pathway enrichment analysis. This analysis aimed to generate hypotheses regarding pathways associated with these proteins, providing preliminary insights into their possible roles in ccRCC development and progression. Using protein expression data from the CPTAC ccRCC dataset, we queried the Reactome Pathway Analysis tool to identify pathways enriched for HEATR1 and SLC27A2. Pathways with a *p*-value < 0.05 were considered statistically significant and biologically relevant. The Reactome enrichment analysis revealed distinct pathways associated with each protein, highlighting their diverse functional roles in ccRCC.

**Figure 4:**
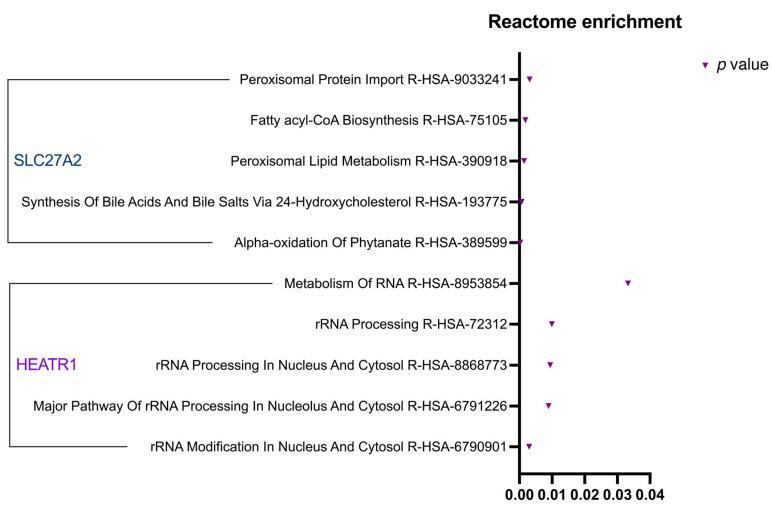
Significant Reactome enrichment pathways for HEATR1 and SLC27A2. Visual representation of statistically significant (*p*-value < 0.05) enriched terms associated with each protein.

The Reactome enrichment analysis (Figure 4) demonstrated that HEATR1 plays a significant part in the processing and modification of rRNA within the nucleus and cytosol. As for SLC27A2, the analysis indicated its key involvement in pathways concerning lipid metabolism and import of proteins the production of bile acids and salts, and the biosynthesis of fatty acyl-CoA. Given the exploratory nature of pathway enrichment tools, these findings serve as hypothesis-generating and require additional experimental validation to clarify their biological significance in ccRCC.

## 4. Discussion

This study examined the levels of HEATR1 and SLC27A2 in ccRCC. These proteins were initially pinpointed using data from the CPTAC database, which included 110 tumor samples. The analysis showed that HEATR1 was significantly increased, while SLC27A2 was significantly decreased in tumor samples compared to normal tissue. IHC analysis in an independent cohort of ccRCC patients, including those with high-grade disease, demonstrated that both HEATR1 and SLC27A2 are expressed in tumor tissues. Although non-malignant tissues were not available for comparison, the observed expression patterns align with the trends reported in the CPTAC dataset. Importantly, elevated HEATR1 and reduced SLC27A2 expression levels were associated with shorter progression-free survival in high-grade ccRCC, supporting their potential as prognostic biomarkers.

IHC analysis revealed diverse subcellular localization of HEATR1 in ccRCC cells, with presence in both the cytoplasm and nucleus, which was previously detected by other studies [12,21]. This observation suggests that HEATR1 may influence various cellular functions, including transcriptional and translational regulation. This hypothesis is supported by Reactome pathway enrichment analysis, which identified significant enrichment in pathways related to RNA metabolism, rRNA processing, and modification processes crucial for cellular homeostasis and often dysregulated in cancer. These findings imply a role for HEATR1 in ribosome biogenesis and protein synthesis, processes commonly altered in cancer cells to sustain their increased growth and proliferation [22]. Previous research has linked HEATR1 to ribosome biogenesis by promoting the nucleolar localization of MYC, a master regulator of ribosome biogenesis and cell growth [22]. This connection is further supported by recent research demonstrating that inhibiting HEATR1 impairs ribosome biogenesis, activating the p53 tumor suppressor pathway and, ultimately, apoptosis in NSCLC cells [23]. While our study is the first to explore HEATR1 in ccRCC, its role in other cancers has been investigated. In line with our findings, HEATR1 is upregulated in various cancer types, where it fuels tumor growth by enhancing ribosome production. For instance, in a previous study, endogenous HEATR1 levels were significantly higher in human cancer cell lines than in primary non-transformed cells [24]. Interestingly, this increased expression was consistently associated with larger nucleoli, a well-established marker of malignancy used by oncopathologists [25]. Additionally, the reported link between HEATR1 depletion and chemoresistance in pancreatic cancer suggests its involvement in critical pathways like Akt and Nrf2, which are known to impact drug sensitivity [21,26]. This suggests a broader biological implication for HEATR1, indicating its potential role in how cells respond to various chemical challenges. Furthermore, HEATR1 might be involved in the heightened ribosome biogenesis that is characteristic of cancer cell proliferation. Understanding its contribution here could provide insights into fundamental cellular processes that are often dysregulated in cancer, like the roles observed for other ribosome biogenesis factors [27]. Its documented influence on drug sensitivity pathways further implies that HEATR1 could play a part in the mechanisms underlying the response of ccRCC cells to chemotherapy [28]. Nevertheless, further mechanistic studies are essential to clarify the precise role of HEATR1 in ccRCC specifically.

SLC27A2, a member of the solute carrier family 27, is a critical fatty acid transporter implicated in various developmental processes, including lipid biosynthesis and fatty acid breakdown. Our Reactome pathway analysis corroborated this function, which showed its association with peroxisomal lipid metabolism and fatty acyl-CoA biosynthesis. Interestingly, SLC27A2′s expression has been linked to obesity development in rat models, suggesting a role in lipid accumulation and weight gain [29]. SLC27A2′s influence extends beyond normal metabolic function; its connection to lipid metabolism is particularly relevant in the context of cancer. Cancer cells often exhibit increased fatty acid uptake and synthesis to fuel their growth and proliferation. Our findings suggest that SLC27A2 may play a role in these metabolic adaptations in ccRCC. While generally considered a tumor suppressor gene in different cancers [30,31], SLC27A2′s role in ccRCC is less clear [32]. Previous research has shown that it is downregulated in ccRCC, potentially contributing to disease progression by promoting epithelial-to-mesenchymal transition (EMT) [33]. Previous studies show that SLC27A2 could be targeted, aiming to either restore its function or to exploit vulnerabilities arising from its reduced expression [34]. In ccRCC, strategies could involve enhancing or re-establishing SLC27A2 function to normalize altered fatty acid metabolism within tumor cells [35]. Inhibitors like Lipofermata, which target FATP2 (the protein encoded by SLC27A2), have demonstrated efficacy in other cancers by disrupting fatty acid uptake and inducing apoptosis, illustrating a principle of targeting the protein’s activity regardless of its expression level [36].

Our Reactome analysis further emphasized SLC27A2′s role in peroxisomal lipid metabolism. Peroxisomes are crucial for maintaining lipid homeostasis, including fatty acid breakdown and lipid synthesis, processes vital for cellular energy balance and signaling [37,38]. Disruptions in their function, potentially caused by SLC27A2 dysregulation, can significantly impact cancer cell behavior [39]. This is supported by findings in other cancers, where SLC27A2 influences processes like fatty acid oxidation (FAO) and interacts with key regulators of lipid metabolism. For example, SLC27A2 is upregulated in colorectal cancer, impacts FAO, and interacts with PPARs, impacting crucial signaling pathways [40]. In endometrial cancer, SLC27A2 is downregulated by FOXM1, affecting fatty acid metabolism and disease progression [41]. In glioblastoma, the altered expression of SLC27 family proteins, including SLC27A2, affects fatty acid uptake and utilization [42]. In ovarian cancer, SLC27A2 regulates a microRNA that targets a drug efflux pump, influencing cisplatin resistance [30]. Recent research in breast cancer has even revealed a role for SLC27A2 in nucleotide metabolism [43]. These findings suggest that in ccRCC, SLC27A2 likely influences disease progression by affecting peroxisomal lipid metabolism and interacting with key regulators like PPARs. While the functional significance remains unclear, this could indicate a role in regulating gene expression or other nuclear processes related to cancer progression. This aligns with findings in differentiated thyroid cancer, where SLC27A2 influenced the C-FOS proto-oncogene expression [44].

Targeting SLC27A2 could disrupt fatty acid metabolism, potentially inhibiting tumor growth and improving patient outcomes. This could involve strategies to modulate SLC27A2 activity, either alone or in combination with other therapies, opening new avenues for targeted treatment of ccRCC. The potential of SLC27A2 as a therapeutic target is further supported by studies in breast cancer, where inhibition of SLC27A2 was shown to suppress proliferation and induce apoptosis in tumor cells [43]. This suggests that strategies to modulate SLC27A2 activity could hold promise for treating various cancers.

This study provides valuable insights into the potential roles of HEATR1 and SLC27A2 in ccRCC, highlighting their association with prognosis. High HEATR1 and low SLC27A2 expression correlated with poorer outcomes, including relapse and metastasis, suggesting their potential utility in risk stratification and treatment decision-making. Further research is needed to validate these findings in larger, more diverse cohorts and to develop robust clinical assays for measuring HEATR1 and SLC27A2 expression. While HEATR1 has been previously implicated in other cancers, its role in ccRCC remains to be fully elucidated. Similarly, the involvement of SLC27A2 in ccRCC, particularly its connection to bile acid metabolism highlighted by our research, is an emerging area of investigation.

This study has several limitations that should be acknowledged. First, the sample size used for IHC validation was relatively small, making it hard to draw robust conclusions. A larger cohort of blinded samples from multiple centers should be included to substantiate the claim that HEATR1 and SLC27A2 are differentially expressed in ccRCC and can be used as prognostic markers. Furthermore, the IHC analysis used research antibodies rather than IVD antibodies. While research antibodies can provide valuable insights, their performance may vary. This limitation should be considered when interpreting the results. Additionally, our study focused primarily on tumor tissues, and corresponding non-malignant samples were not available in the cohort. Future research should focus on elucidating the precise mechanisms by which HEATR1 and SLC27A2 contribute to ccRCC development and progression. Investigating the functional significance of nuclear SLC27A2 and exploring the connection between SLC27A2 and bile acid metabolism are also important areas for future research. Furthermore, pre-clinical and clinical studies are needed to evaluate the therapeutic potential of targeting HEATR1 and SLC27A2 in ccRCC.

## 5. Conclusions

In conclusion, our study identified HEATR1 and SLC27A2 as potential prognostic markers in ccRCC. Elevated HEATR1 and reduced SLC27A2 expression were significantly associated with shorter progression-free survival in high-grade ccRCC patients, aligning with their differential expression in CPTAC data. These findings provide new insights into the molecular mechanisms underlying ccRCC and highlight the importance of further research to translate these discoveries into clinical applications. Nonetheless, additional experiments and more comprehensive data should be used to validate the study’s results and conclusions.

## Figures and Tables

**Table 1:** Primary antibodies used for immunohistochemistry.

Antibody	Source	Identifier	Clone	Dilution
Rabbit polyclonal anti-HEATR1	Bioss (Woburn, MA, USA)	Cat# bs-15438R, RRID:AB_2934056	Polyclonal	1:200
Rabbit polyclonal anti-SLC27A2	Atlas Antibodies (Stockholm, Sweden)	Cat# HPA026089, RRID:AB_1857060	Polyclonal	1:500

**Table 2:** Classification of IHC specimens based on H-scores.

Protein	H-Score Range—High IHC Score Group	H-Score Range—Low IHC Score Group	Reaction
HEATR1	110–260, *n* = 25	0–105, *n* = 26	Nuclear, cytoplasmic, membranous
SLC27A2	120–260, *n* = 31	0–100, *n* = 21	Cytoplasmic, membranous, nuclear

**Table 3:** HR and 95% CIs for HEATR1 and SLC27A2, evaluating their impact on PFS and OS in patients’ survival in the ccRCC validation cohort, with protein expression measured by IHC.

	Progression-Free Survival (PFS)		Overall Survival (OS)	
	Hazard Ratio (HR)	95% CI of HR	Hazard Ratio (HR)	95% CI of HR
HEATR1	5.141	1.689–15.64	5.636	2.018–15.74
SLC27A2	3.299	1.374–7.921	3.798	1.31–11.01

## Data Availability

All data on which the manuscript was based, if not already included in its text, are available from the corresponding author on a reasonable request.

## Supplementary Materials

The following supporting information can be downloaded at: https://www.mdpi.com/article/10.3390/cancers17132234/s1, Table S1. Characteristics of patients included in the study.
