# Supplementary material for: Immunohistological Examination of HEATR1 and SLC27A2 Expression in ccRCC Samples to Evaluate Their Potential as Prognostic Markers—A Preliminary Study
Source: Cancers (Basel). 2025 Jul 3;17(13):2234. doi: 10.3390/cancers17132234 (PMC12249168; doi:10.3390/cancers17132234)
Supplement: Supplementary file 1 [file cancers-17-02234-s001.zip › cancers-3595241-supplementary.pdf]

No.	Case ID	Case included in the CPTAC CCRCC discovery cohort	Date of primary tumor surgery	Date of metastasis appearance	Age at primary tumor surgery	Alive or Date of death	Histology grade (G)	Metastasis location	Number of days from the date of primary tumor surgery to the date of metastasis appearance or last contact when no metastasis developed - PFS	Metastasis - yes (1) or no (0)	Number of days from the date of primary tumor surgery to the date of death or last contact when patient alive - OS	Patient status - deceased (1) or alive (0)
1	C3N-00305	Yes	09-maj-16	11-sie-21	60	Alive	G3	No metastasis	1920	0	2226	0
2	C3N-00310	Yes	17-maj-16	09-lis-20	84	09-lis-20	G1	No metastasis	1637	0	1637	1
3	C3N-00312	Yes	31-maj-16	11-sie-21	68	Alive	G2	No metastasis	1898	0	2204	0
4	C3N-00313	Yes	10-cze-16	11-sie-21	31	Alive	G3	No metastasis	1888	0	2194	0
5	C3N-00314	Yes	16-cze-16	01-lip-16	78	31-sie-16	G2	Liver	15	1	140	1
6	C3N-00315	Yes	28-cze-16	11-sie-21	68	Alive	G2	No metastasis	1870	0	2176	0
7	C3N-00320	Yes	12-lip-16	11-sie-21	67	Alive	G3	No metastasis	1856	0	2162	0
8	C3N-00437	Yes	18-sie-16	17-lut-21	69	17-lut-21	G2	No metastasis	1644	0	1644	1
9	C3N-00491	Yes	01-wrz-16	22-gru-16	54	22-gru-16	G3	No metastasis	112	0	113	1
10	C3N-00492	Yes	05-wrz-16	11-sie-21	49	Alive	G2	No metastasis	1801	0	2107	0
11	C3N-00494	Yes	13-wrz-16	11-sie-21	66	Alive	G3	No metastasis	1793	0	2099	0
12	C3N-00831	Yes	30-wrz-16	07-lis-16	52	29-gru-17	G2	Lungs	38	1	455	1
13	C3N-00832	Yes	28-wrz-16	11-sie-21	79	Alive	G2	No metastasis	1778	0	2084	0
14	C3N-00834	Yes	07-paź-16	11-sie-21	65	Alive	G2	No metastasis	1769	0	2075	0
15	C3N-01175	Yes	21-lis-16	11-sie-21	60	Alive	G1	No metastasis	1724	0	2030	0
16	C3N-01176	Yes	21-lis-16	05-gru-16	71	Alive	G2	Lungs	14	1	2030	0
17	C3N-01178	Yes	29-lis-16	11-sie-21	58	Alive	G2	No metastasis	1716	0	2022	0
18	C3N-01179	Yes	13-gru-16	11-gru-21	72	Alive	G2	No metastasis	1824	0	2008	0
19	C3N-01180	Yes	13-gru-16	11-gru-21	51	Alive	G2	No metastasis	1824	0	2008	0
20	C3N-01361	Yes	19-gru-16	12-gru-21	48	Alive	G2	No metastasis	1819	0	2002	0
21	C3N-01648	Yes	06-lut-17	11-sie-21	69	Alive	G2	No metastasis	1647	0	1953	0
22	C3N-01649	Yes	7-lut-17	16-lut-17	51	Alive	G2	Liver	9	1	1952	0
23	C3N-01651	Yes	09-lut-17	06-lut-18	58	Alive	G2	Lung	362	1	1950	0
24	C3N-01654	No	13-mar-17	15-lip-20	64	Alive	G3	Liver	1220	1	1918	0
25	C3N-01655	No	22-mar-17	01-sie-19	64	Alive	G2	Kidney	862	1	1909	0
26	C3N-01656	No	04-kwi-17	01-paź-18	68	Alive	G2	Lung	545	1	1896	0
27	C3N-01657	No	05-kwi-17	11-sie-21	74	Alive	G2	No metastasis	1589	0	1895	0
28	C3N-01905	No	27-kwi-17	11-sie-21	59	Alive	G1	No metastasis	1567	0	1873	0
29	C3N-02262	No	13-maj-17	04-paź-17	67	Alive	G2	Lung	144	1	1857	0
30	C3N-02263	No	12-maj-17	11-sie-21	69	Alive	G2	No metastasis	1552	0	1858	0
31	C3N-02264	No	16-maj-17	30-maj-17	68	Alive	G2	Kidney	14	1	1854	0
32	C3N-02266	No	22-maj-17	11-sie-21	56	Alive	G1	No metastasis	1542	0	1848	0
33	C3N-03018	No	13-wrz-17	11-sie-21	65	Alive	G2	No metastasis	1428	0	1734	0
34	C3N-03019	No	13-wrz-17	11-sie-21	46	Alive	G2	No metastasis	1428	0	1734	0
35	C3N-03020	No	19-wrz-17	18-sty-21	65	18-sty-21	G1	No metastasis	1217	0	1217	1
36	C3N-03021	No	21-wrz-17	11-sie-21	50	Alive	G3	No metastasis	1420	0	1726	0
37	4585-97/19	No	14-mar-19	14-mar-19	62	Alive	G3	Lungs	0	1	1187	0
38	5207-18/19	No	1-kwi-19	19-sty-21	50	Alive	G3	Lungs	659	1	1169	0
39	3164-70/14	No	4-listopad-14	15-maj-15	62	15-sty-16	G3	Lungs	192	1	437	1
40	6184-87/13	No	03-gru-13	09-kwi-14	42	26-kwi-14	G2	Lungs, liver, kidney	127	1	144	1
41	16/02/9100	No	7-lip-16	28-wrz-17	80	30-gru-17	G2	Lungs, liver, kidney	448	1	541	1
42	3321-27/13	No	25-wrz-13	26-sie-14	58	23-lis-14	G1	Lungs, liver, kidney	335	1	424	1
43	8034-43/13	No	24-maj-13	12-lip-13	56	15-sie-17	G3	Lungs	49	1	1544	1
44	2652-59/14	No	26-lut-14	19-sty-15	54	Alive	G1	Lungs, kidney	327	1	2878	0
45	1944-50/15	No	8-paź-15	18-wrz-15	52	17-sty-16	G3	Liver, bones	0	1	101	1
46	6072-76/16	No	04-maj-16	08-lis-11	57	27-wrz-18	G2	Lungs	0	1	876	1
47	6512-21/15	No	2-cze-15	13-wrz-18	55	Alive	G3	Lungs	1199	1	2309	0
48	0758-61/15	No	16-wrz-15	09-cze-21	56	Alive	G2	Kidney	2093	1	2462	0
49	3980-89/15	No	23-lis-15	04-paź-17	50	Alive	G2	Lungs	681	1	2394	0
50	8947-54/16	No	5-lip-16	25-sie-16	57	30-paź-19	G3	Lungs	51	1	1212	1
51	3086-94/13	No	2-lip-15	24-kwi-17	51	02-lip-17	G3	Bones	662	1	731	1
52	4119-28/18	No	11-paź-18	03-kwi-19	52	Alive	G2	Bones	174	1	1341	0
